# Consistency of the S5 DNA methylation classifier in formalin‐fixed biopsies versus corresponding exfoliated cells for the detection of pre‐cancerous cervical lesions

**DOI:** 10.1002/cam4.3849

**Published:** 2021-03-12

**Authors:** Caroline Reuter, Matthew Preece, Rawinder Banwait, Sabrina Boer, Jack Cuzick, Attila Lorincz, Belinda Nedjai

**Affiliations:** ^1^ Centre for Cancer Prevention Wolfson Institute of Preventive Medicine Queen Mary University of London London UK; ^2^ Department of Urology Radboud University Medical Center Radboud Institute for Molecular Life Sciences Nijmegen the Netherlands

**Keywords:** biomarker, cervical cancer, DNA methylation, formalin‐fixed paraffin‐embedded samples (FFPE), S5 classifier

## Abstract

Methylation biomarkers are promising tools for diagnosis and disease prevention. The S5 classifier is aimed at the prevention of cervical cancer by the early detection of cervical intraepithelial neoplasia (CIN). S5 is based on pyrosequencing a promoter region of *EPB41L3* and five late regions of HPV types 16, 18, 31, and 33 following bisulfite conversion of DNA. Good biomarkers should perform well in a variety of sample types such as exfoliated cells, fresh frozen or formalin‐fixed paraffin‐embedded (FFPE) materials. Here, we tested the performance of S5 on 315 FFPE biopsies with paired exfoliated cervical samples using four different conversion kits (Epitect Bisulfite, Epitect Fast Bisulfite, EZ DNA Methylation, and EZ DNA Methylation‐Lightning). The S5 values from FFPE biopsies for all kits were significantly correlated with those obtained from their paired exfoliated cells. For the EZ DNA Methylation kit, we observed an average increased methylation of 4.4% in FFPE. This was due to incomplete conversion of DNA (73% for FFPE vs. 95% for cells). The other kits had a DNA conversion rate in FFPE similar to the cells (95%–97%). S5 performed well at discriminating <CIN2 lesions from CIN2+ lesions on the FFPE with all kits given optimized adjustments to the cut‐off. The area under the curve (AUC) for S5 on FFPE was not significantly different from the paired cells (0.74–0.79 vs. 0.81). The best sensitivity and specificity were obtained for EZ DNA Methylation after the adjustment of the cut‐off to reflect its lower conversion rate. Consistent methylation results can be obtained from FFPE material regardless of the conversion kit used. The S5 classifier performed as well on FFPE material as on exfoliated cells with adjusted cut‐off allowing easier clinical implementation.

## Graphical abstract

The S5 Classifier is a DNA methylation‐based assay designed to detect pre‐cancerous cervical tissue. It was designed using a particular bisulfite conversion kit and optimized on fresh cells only. In this study, we establish that the S5 Classifier works to a similar level of sensitivity and specificity in FFPE tissue as it does in exfoliated samples and with three other bisulfite conversion kits. This validates its validity as an effective biomarker assay.

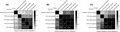

## 1 INTRODUCTION

Cervical cancer is caused by a persistent infection with high‐risk human papillomaviruses (hrHPV). If identified early, high‐grade cervical intraepithelial neoplasias (CIN grades 2 and 3) can be effectively treated to prevent their development into cancer. In cervical cancer, methylation biomarkers are promising diagnostic and prognostic tools that could be used for prevention in clinical settings.^1^, ^2^, ^3^ DNA methylation is an epigenetic mechanism used to regulate gene expression in cells and is an essential process for cell differentiation and embryonic development. Aberrant DNA methylation has been linked to various diseases including cancer.^4^, ^5^ One of the most accurate methods of measuring DNA methylation is the bisulfite conversion of unmethylated cytosines to thymidines followed by pyrosequencing.^6^ Our team has developed and validated a pyrosequencing methylation classifier for detecting cancer and pre‐cancerous lesions (CIN2+) from exfoliated cervical cells.^3^, ^7^, ^8^, ^9^, ^10^ The S5 classifier comprises targets in the promoter region of tumor suppressor gene *EPB41L3* and late viral regions of HPV16, HPV18, HPV31, and HPV33. Good biomarkers should have a high sensitivity and specificity, good positive predictive value, and perform well in different sample types such as exfoliated cells, fresh, frozen or formalin‐fixed paraffin‐embedded (FFPE) biopsies.^11^ When the S5 classifier was developed it was performed so using exfoliated cells and the Zymo EZ DNA Methylation kit. To further establish the S5 classifier as a high‐value biomarker it is the key to ensure its validity using both FFPE and a range of bisulfite conversion kits. Establishing the viability of the S5 classifier in FFPE samples also allows it to be used in retrospective studies using archived samples. In a broader context if the S5 classifier is shown to work with FFPE material and multiple conversion kits, there are implications for epigenetic studies using FFPE material. DNA extracted from FFPE material generally shows lower quality and amount than from fresh or frozen material. Tissue fixation in buffered formalin causes inter‐strand DNA or protein–DNA crosslinks, fragmentation, deamination of cytosines, and conformational change in the DNA.^12^ Therefore, bisulfite conversion efficacy can be a problem on FFPE material, possibly leading to unreliable results.^13^ Although assays such as genotyping, copy number or DNA methylation performed on FFPE samples usually have a lower success rate than on fresh or frozen material,^14^ they can still provide adequate information for epidemiological studies and to inform clinical decisions. In this study, we used a set of paired FFPE and exfoliated cell samples from the same women to test the performance of the S5 classifier at detecting CIN2+ in samples fixed in formalin. We compared four bisulfite conversion kits, the Zymo EZ DNA Methylation kit (EZ Std), the Zymo EZ DNA Methylation‐Lightning kit (EZ Lightning), the Qiagen Epitect Bisulfite kit (Epitect Std), and the Qiagen Epitect Fast Bisulfite kit (Epitect Fast). We discuss differences between the two sample types and the four bisulfite conversion kits in terms of conversion efficiency and methylation levels obtained for the S5 classifier. We report area under the ROC curve (AUC), sensitivity, and specificity with confidence intervals.

## 2 MATERIALS AND METHODS

We tested the performance of a risk score, called the S5 classifier, at detecting pre‐cancerous (CIN2 n = 41 and CIN3 n = 105) and cancerous (n = 3) cervical intraepithelial neoplasia (CIN2+) on formalin‐fixed paraffin‐embedded (FFPE) biopsies collected at colposcopy. The results were compared to those obtained from a set of paired exfoliated cell samples (reference standard) from the same women. We tested four different bisulfite conversion kits: The Zymo EZ DNA Methylation kit (EZ Std) which was the kit used to develop the S5 classifier, the Zymo EZ DNA Methylation‐Lightning (EZ Lightning), the Qiagen Epitect Bisulfite Kit (Epitect Std), and the Qiagen Epitect Fast Bisulfite Kit (Epitect Fast).

### 2.1 Study population and clinical specimens

Ethical approval and consent to participate were given by the Hammersmith and Queen Charlotte's & Chelsea Research Ethics Committee (Ethics no. 05/Q0406/57). Paired samples consisting of FFPE biopsies and exfoliated cells from liquid‐based cytology specimens (ThinPrep; Hologic, Bedford, USA) both taken at colposcopy were obtained from archived material from the Predictors studies collected from 2005 to 2009.^15^, ^16^ The women in both studies were selected from a population of women who had been referred to colposcopy. Women were referred to colposcopy if they had one abnormal smear. The selection of women had an age range of 17–72 (median age 29) and their HPV status was unknown at the time of referral. Final histopathological diagnoses were based on reviews by at least two pathologists. The highest grade of abnormality seen in the biopsy was used.

### 2.2 DNA extraction from cells in ThinPrep

DNA was extracted from aliquots of the liquid‐based cytology samples with the QIAamp DNA Mini Kit (Qiagen Inc., Hilden, Germany) following the manufacturer's instructions with the exception of an additional first step. In the additional step, the cells were washed in 1 ml of PBS, centrifuged at 13,000 rpm for 1 minute, the supernatant discarded, and 200 µl of PBS added before the lysis step.

### 2.3 DNA extraction from FFPE biopsies

For the first 59 biopsies, twelve 5 μm FFPE sections were cut on a microtome using a new blade for each block. Sections were stored at −70°C until DNA extraction. Entire sections were scraped from the slides using a scalpel blade and deparaffinized using three washes in xylene and one wash with 100% ethanol. DNA was extracted using QIAamp DNA FFPE Tissue Kit (QIAGEN) according to manufacturer's instructions with an initial incubation step at 56°C for 16–18 hours with proteinase K and a 1‐hour incubation at 90°C. The DNA concentration was measured using a NanoDrop 1000 Spectrophotometer (Thermo Fisher Scientific).

The remaining 256 biopsies were extracted using a different method to try and maximize DNA recovery.^17^ Ten 5 µm FFPE sections were cut using a new blade for each block and directly put in a tube and stored at −70°C until DNA extraction. One hundred and sixty microliters of hexadecane were added to each tube, followed by a 5‐minute incubation at 56°C. Two hundred microliters of universal extraction buffer containing 50 mM Tris–HCl pH 8.0, 1 mM EDTA, and 0.05% SDS^18^ were added to the tube along with 400 µg of Proteinase K (Qiagen) and incubated overnight at 56°C followed by a 1‐hour incubation at 90°C. The lower phase was then transferred to a new tube and stored at −20°C before PCR. The DNA concentration was measured using a Qubit Fluorometer with the dsDNA HS Assay Kit (Thermo Fisher Scientific, Waltham, MA, USA).

### 2.4 Bisulfite conversion and DNA methylation assays

The DNA obtained from exfoliated cells was bisulfite converted using the EZ DNA Methylation kit (Zymo Research, CA, USA, abbreviated as EZ Std) using 200 ng of DNA and following the manufacturer's instructions.

The FFPE material was bisulfite converted using four different kits: the EZ Std, the EZ DNA Methylation‐Lightning kit (Zymo Research, CA, USA, abbreviated as EZ Lightning), the Epitect Bisulfite kit (Qiagen, Hilden, Germany, abbreviated as Epitect Std), and the Epitect Fast Bisulfite Kit (Qiagen, Hilden, Germany, abbreviated as Epitect Fast). All kits were used following the manufacturer's instructions with an input of 200 ng in 40 μl of DNA for EZ Std, Epitect Std, and Epitect Fast and 200 ng in 20 μl (maximum recommended volume) for EZ Lightning. Differences between these kits are highlighted in Supp. Table S1.

We used previously optimized PCR conditions for the markers included in the S5 classifier.^1^, ^7^, ^19^, ^20^, ^21^ The S5 classifier comprises CpGs in the promoter region of EPB41L3 (CpG sites 425, 427, and 438 relative to transcription start site) and viral regions of HPV16 (L1: CpG sites 6367, 6389 and L2: CpG sites 4238, 4247, 4259 4268, 4275), HPV18 (L2: CpG sites 4257, 4262, 4266, 4269, 4275, 4282), HPV31 (L1: CpG sites 6352 and 6354), and HPV33 (L2: CpG sites 5557, 5560, 5566 and 5572). Amplifications were performed using the PyroMark PCR kit (QIAGEN, Germany) with 10 ng of converted DNA (except for HPV18_L2_ PCR, for which 20 ng of DNA was used) in a 25µL volume with final reagent concentrations of 0.2 µM for PCR primers, 1x Coral Load, and 1x PyroMark mix. PCR cycling conditions were 15 minutes at 94°C, followed by 45 cycles of 94°C, 54°C (51°C for HPV16_L2_), 72°C each for 30 seconds, and a final extension at 72°C for 10 minutes. The PCR products were pyrosequenced using a PyroMark™Q96 ID (Qiagen) instrument as previously described.^22^ All pyrosequencing runs included positive controls of known methylation level (0%, 50%, and 100%) to allow standardized direct comparisons between different primer sets and a negative control.

### 2.5 Calculating the S5 classifier

The S5 classifier was developed and validated on cells collected in liquid‐based cytology medium.^3^, ^21^ S5 is defined as the sum of six methylation components: S5 = 30.9(*EPB41L3*) + 13.7(HPV16_L1_) + 4.3(HPV16_L2_) + 8.4(HPV18_L2_) + 22.4(HPV31_L1_) + 20.3 (HPV33_L2_). All components, except HPV16_L2_, are calculated as the percentage of average methylation of the CpG investigated (3 for EPB41L3, 2 for HPV16_L1_, 6 for HPV18_L2_, 2 for HPV31_L1_, and 3 for HPV33_L2_). For HPV16_L2_, the proportion of 3 CpGs (sites: 4238, 4259, 4275) with methylation values >0 is used.^7^

### 2.6 Bisulfite conversion efficiency

The efficiency of bisulfite conversion was measured by an in‐house real‐time PCR using two pairs of primers (“converted” and “unconverted”) targeting a region of the β‐actin gene containing no CpG sites. After a 100% efficient bisulfite conversion, all the cytosines should be fully converted and anneal to the “converted” primers F and R. If some cytosines are left unconverted, the DNA strand will anneal to the “unconverted” primers F and R. The “converted” primer pair anneals to the fully converted DNA (Forward primer: 5′‐TGGTGATGGAGGAGGTTTAGTAAGT‐3′, Reverse primer: 5′‐AACCAATAAAACCTACTCCTCCCTTAA‐3′), the “unconverted” primer pair to unconverted DNA (Forward primer: 5′‐TGGTGATGGAGGAGGCTCAGCAAGT‐3′, Reverse primer: 5′‐AGCCAATGGGACCTGCTCCTCCCTTGA‐3′). The samples were run in duplicate on a Quantstudio5 (Thermo Fisher Scientific) using a 384‐well block. For each PCR reaction, 1.3 μl of bisulfite‐converted DNA (diluted 1:5), 6.3 μl 2x KAPA SYBR FAST qPCR Master Mix, 0.6 μl each forward and reverse primer (final concentrations of 0.5 μM), and 4.4 μl molecular‐grade water were used. Real‐time PCR conditions were 95°C for 10 minutes followed by 40 cycles of 95°C for 15 seconds, 60°C for 1 minutes with data acquisition after each cycle. In the end, a dissociation curve was added ranging from 60°C to 95°C. The mean of the duplicate CT values was calculated for the converted primer and unconverted primer values. The delta CT was calculated as the average of “converted” CT minus the average of “unconverted” value and the percentage of “unconverted” calculated as 100%/ (1 + (2^ (delta Ct). The conversion efficiency was calculated as 100% minus % “unconverted.”

### 2.7 Statistical analysis

The correlations between the S5 classifier obtained from FFPE biopsies and cells were computed using the Spearman r coefficients. A Wilcoxon matched‐pairs signed‐rank test was used to assess the difference in S5 methylation values between the paired FFPE biopsies and exfoliated cells samples. The performance of the S5 classifier was calculated using CIN2/3 as the primary clinical endpoint. The ability of S5 to separate <CIN2 from CIN2+ samples was assessed with Mann–Whitney tests and ROC curves (AUC are given with 95% confidence intervals). The differences between the AUC of the ROC curves from exfoliated cells and FFPE biopsies were tested using DeLong's test. The pre‐defined cut‐off for S5 on exfoliated cells was 0.8^7^ and was used to calculate sensitivity and specificity of S5 on the FFPE biopsies, but we also calculated these performance indicators on retrospectively adjusted cut‐offs. Reproducibility of the assays was evaluated graphically using Bland–Altman plots. Statistical analyses were conducted using GraphPad Prism and R.

## 3 RESULTS

**FIGURE 1:**
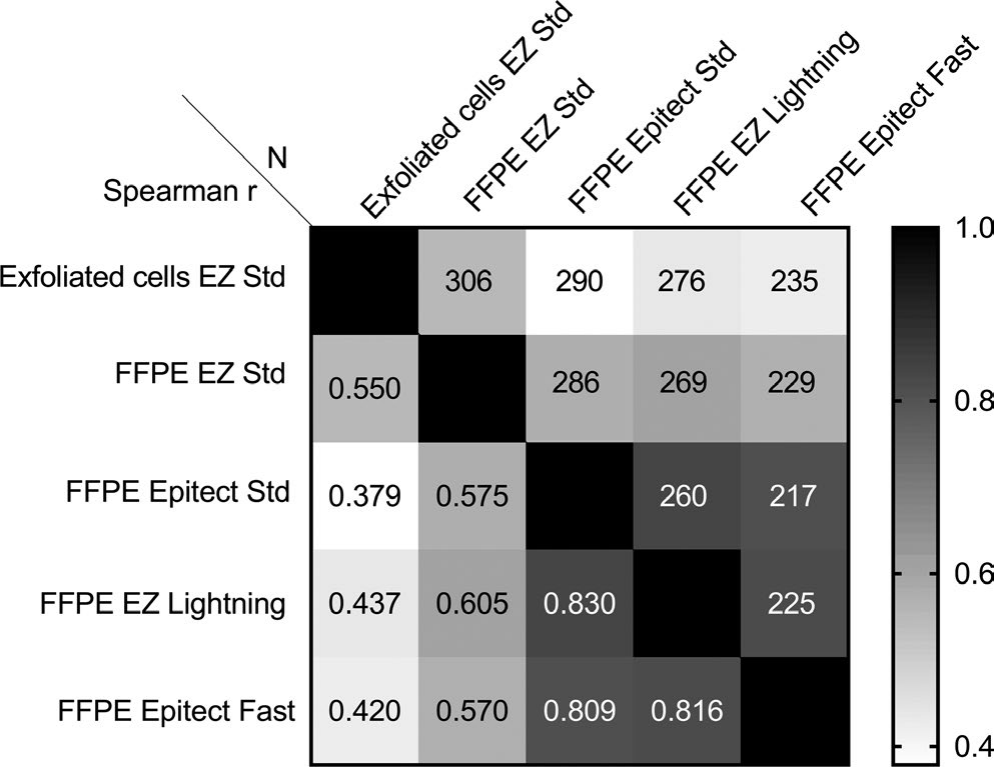
Correlation of S5 classifier methylation levels obtained from exfoliated cells and FFPE material. DNA from the exfoliated cells was converted with the Zymo EZ DNA Methylation kit (EZ Std). Four bisulfite conversion kits were used on the FFPE material: EZ Std, Zymo EZ DNA Methylation‐Lightning (EZ Lightning), Qiagen Epitect Bisulfite kit (Epitect Std) and Qiagen Epitect Bisulfite Fast kit (Epitect Fast). The correlation coefficients are given below the diagonal and the number of samples in the comparisons above. All the correlations were statistically significant (*p* < 0.0001, Spearman correlation tests)

We tested the performance of the S5 methylation classifier and efficiency of bisulfite conversion on 315 paired samples consisting of FFPE biopsies and exfoliated cell samples. In total, there were 166 <CIN2 (40 Normal and 126 CIN1) and 149 CIN2+ samples (41 CIN2 and 105 CIN3 and 3 cancers). We tested four bisulfite conversion kits on DNA extracted from the FFPE material and compared results to their paired exfoliated samples bisulfite converted with the EZ DNA Methylation kit (EZ Std). In total, we tested 1422 samples: 315 exfoliated cell samples bisulfite converted with EZ Std, 306 FFPE bisulfite converted with EZ Std, 290 FFPE converted with Epitect Std, 276 FFPE converted with EZ Lightning, and 235 FFPE converted with Epitect Fast (Figure 1.).

### 3.1 Methylation levels of FFPE biopsies compared to exfoliated cells

**FIGURE 2:**
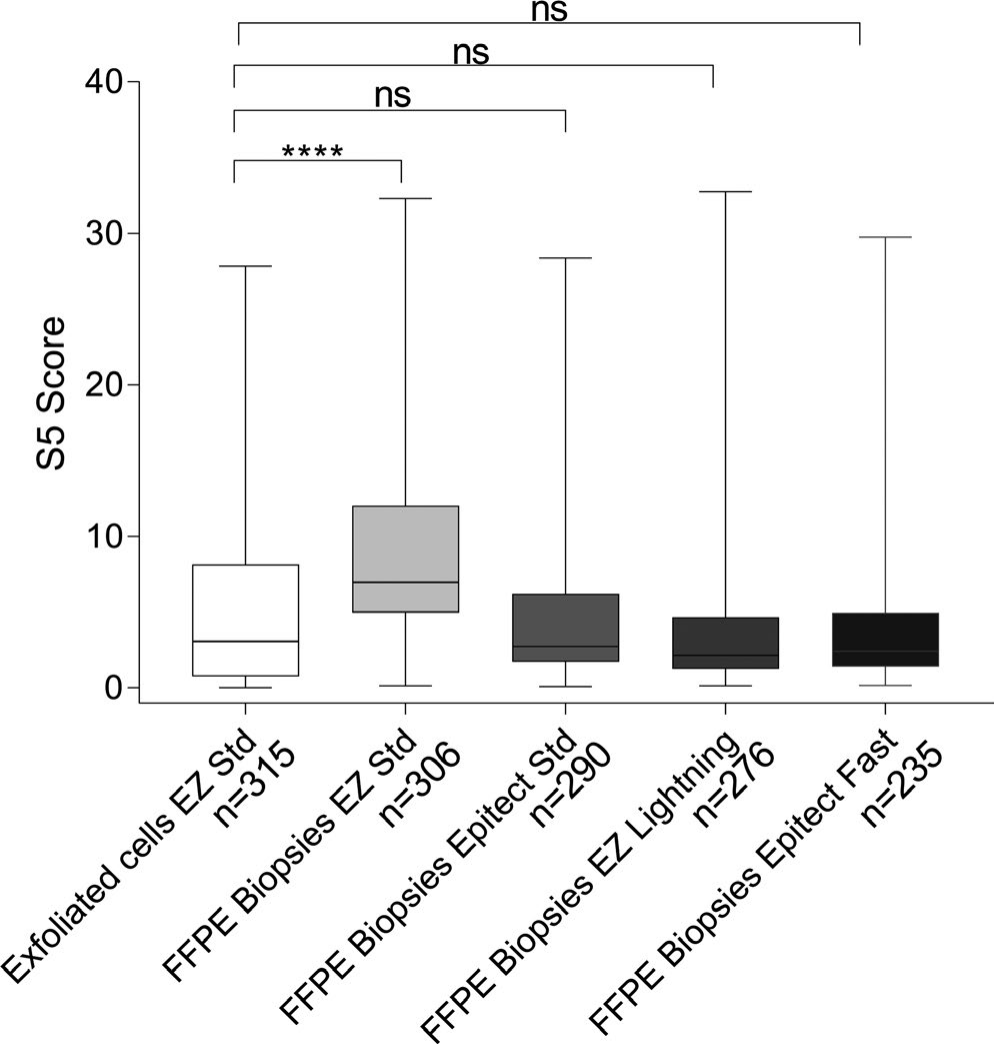
Boxplot of S5 classifier methylation levels comparing results obtained from exfoliated cell‐DNA (reference standard) bisulfite converted with EZ DNA Methylation kit (EZ Std) and the four bisulfite conversion kits used on FFPE biopsies. These kits were the EZ Std, EZ DNA Methylation‐Lightning kit (EZ Lightning), the Epitect Bisulfite kit (Epitect Std) and the Epitect Fast Bisulfite Kit (Epitect Fast). The S5 values obtained using the EZ Std kit on FFPE material were significantly higher than those from exfoliated cells. There were no significant differences between the S5 values in the reference exfoliated cells and the other three bisulfite conversion kits. *: *p* < 0.05, **: *p* < 0.001, ***: *p* < 0.0005, ****: *p* < 0.0001. Only significant comparisons are represented. The top of boxes represents the upper quartile, bottom the lower quartile and line the median. The whiskers expand to the minimum and maximum values

**FIGURE 3:**
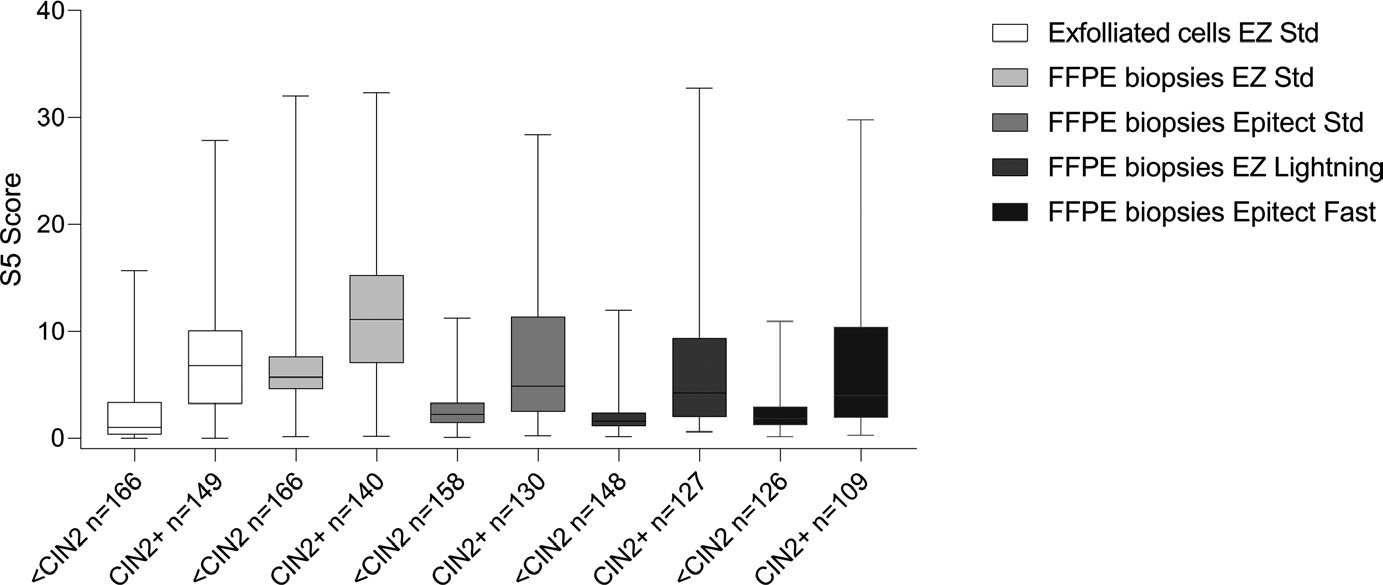
Boxplot of S5 classifier methylation levels between <CIN2 and CIN2+. The S5 score was significantly higher in CIN2+ than in <CIN2 for the exfoliated cells and FFPE material using all four bisulfite conversion kits (*p* < 0.0001, Mann‐Whitney tests). Higher absolute methylation levels were obtained with the EZ DNA Methylation kit (EZ Std) compared to exfoliated cells with the same kit and FFPE material with the other three kits Epitect Std: Epitect Bisulfite kit; EZ Lightning: EZ DNA Methylation‐Lightning; Epitect Fast: Epitect Bisulfite Fast kit. The top of boxes represents the upper quartile, bottom the lower quartile and line the median. The whiskers expand to the minimum and maximum values

The S5 methylation values from FFPE biopsies for the four bisulfite conversion kits were significantly correlated with those obtained from the exfoliated cells (Spearman r ranged from 0.379 to 0.550, *p* < 0.0001, Figure 1.). High methylation values obtained in biopsies were also mostly high in exfoliated cells. In the FFPE material, the highest correlations were obtained between the Epitect Std and EZ Lightning (r = 0.830, *p* < 0.0001), the Epitect Std and Epitect Fast (r = 0.809, *p* < 0.0001), and the EZ Lightning and Epitect Fast (r = 0.816, *p* < 0.0001). The S5 classifier values were significantly higher in FFPE biopsies bisulfite converted with the EZ Std kit compared to their paired exfoliated cell samples (Wilcoxon matched‐pairs signed‐rank tests, *p* < 0.0001, Figure 2. Table 1). On average for the paired samples, S5 was higher by 4.4% in the FFPE biopsies converted with EZ Std compared to their paired cell specimens. The three other bisulfite conversion kits produced methylation results on FFPE samples that were not significantly different from the exfoliated cells (Figure 3. and Table 1).

**TABLE 1:** Descriptive statistics of the S5 methylation pyrosequencing score for exfoliated cells and FFPE biopsies using four different bisulfite conversion kits

	S5 exfoliated cells EZ Std	S5 FFPE biopsies EZ Std	S5 FFPE biopsies Epitect Std	S5 FFPE biopsies EZ Lightning	S5 FFPE biopsies Epitect Fast
Number of samples	315	306	290	276	235
Minimum	0.00	0.14	0.00	0.00	0.15
Maximum	27.84	32.30	28.38	32.73	29.60
Mean	4.86	9.11	4.71	4.11	4.38
Std deviation	5.02	5.77	4.95	4.74	4.80
Wilcoxon matched‐pairs signed rank test p‐values (n)^a^	Reference	<0.0001 (306)	0.2827 (288)	0.9592 (275)	0.5701 (235)

^a^ Bonferoni correction, α = 0.0125.

### 3.2 Methylation levels of individual S5 genes of FFPE biopsies compared to exfoliated cells

**FIGURE 4:**
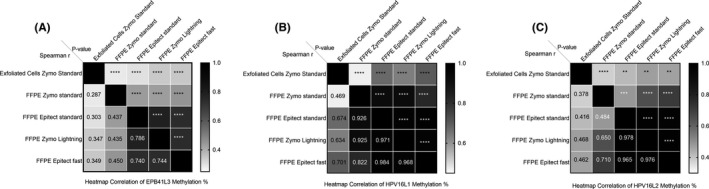
Heatmap of the correlation of A. EPB41L3, B. HPV16L1 and C. HPV16L2 gene methylation levels obtained from exfoliated cells and FFPE material. DNA from the exfoliated cells was converted with the Zymo EZ DNA Methylation kit (Exfoliated Cells Zymo Standard). Four bisulfite conversion kits were used on the FFPE material: Zymo EZ DNA Methylation (FFPE Zymo Standard), Qiagen Epitect Bisulfite kit (FFPE Epitect Standard), Zymo EZ DNA Methylation‐Lightning (FFPE Zymo Lightning) and Qiagen Epitect Bisulfite Fast kit (FFPE Epitect Fast). The Spearman's correlation coefficients are given below the diagonal and p‐values above (*: *p* < 0.05, **: *p* < 0.01, ***: *p* < 0.001, ****: *p* < 0.0001)

Methylation levels for EPB41L3 were significantly correlated between exfoliated cells and all kits (*p* < 0.0001), Spearman ranged from 0.287 to 0.786. The highest correlations were between Epitect Std and Zymo Lightning (r = 0.786), the Epitect Std and Epitect Fast (r = 0.740), and the EZ Lightning and Epitect Fast (r = 0.744) (Figure 4). Methylation levels for HPV16L1 were all significantly correlated (*p* < 0.0001) with correlation between the kits ranging from Spearman r 0.822 to 0.984. Correlation between the exfoliated cells was lower with Spearman r from 0.469 to 0.701 but still significant (*p* < 0.0001) (Figure 4). Correlation between the kits and exfoliated cells for HPV16L2 was all significant (*p* < 0.01‐*p* < 0.0001) with the highest correlations between Epitect Std and Zymo Lightning (r = 0.978, *p* < 0.0001), the Epitect Std and Epitect Fast (r = 0.965, *p* < 0.0001), and the EZ Lightning and Epitect Fast (r = 0.976, *p* < 0.0001) (Figure 4). Due to the lower prevalence of HPV18, 31, and 33, it was not possible to calculate correlations between all kits and not all calculated Spearman r values were significant. For HPV18 exfoliated cells versus Zymo Std (*p* < 0.05), Epitect Std (*p* < 0.05), and Zymo Lightening (*p* < 0.01) were significant with Spearman r ranging from 0.517 to 0.929 (Supp. Figure S1). All correlations between Epitect Std, Zymo Lightening, and Epitect fast were significant (*p* < 0.001) and r = 1.000 for all 3. For HPV31, only Zymo Std and exfoliated cells had sufficient data to calculate the correlation (r = 0.615 *p* < 0.05) (Supp. Figure S1). In HPV33, the only significant correlations were between exfoliated cells and Zymo Std (r = 0.711. *p* < 0.0001) and Zymo Std and Epitect Std (r = 0.800, *p* < 0.05) (Supp. Figure S1).

### 3.3 Bisulfite conversion rate and assay repeatability

**FIGURE 5:**
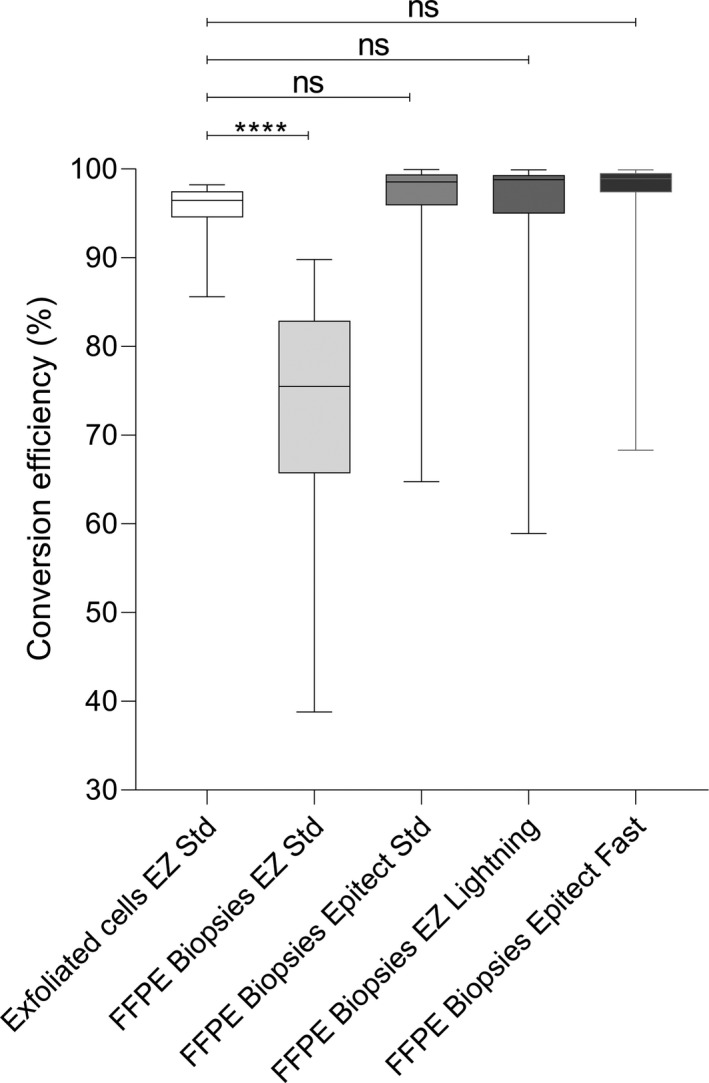
Comparison of bisulfite conversion efficiency between exfoliated cells and FFPE material. The top of boxes represents the upper quartile, bottom the lower quartile and line the median. The whiskers expand to the minimum and maximum values. The Epitect Standard, Epitect Fast and EZ Lightning kits performed well and their rates of conversion were not significantly different from the exfoliated cells. The EZ DNA Methylation kit (EZ Std) showed a lower conversion rate than the exfoliated cells (*p* < 0.0001, Wilcoxon matched‐pairs signed rank test)

We investigated the reason for the higher methylation values in FFPE samples bisulfite converted with the EZ Std kit using an in‐house real‐time PCR methylation‐specific assay targeting the β‐actin gene (Supp. Table S2 and Figure 5.). We found that the bisulfite conversion rate of exfoliated cells was on average 95% using the EZ Std kit; however, this kit only had a conversion rate of 73% for FFPE material (*p* < 0.0001). The other kits on FFPE samples had very good conversion rates that were not significantly different from the exfoliated cells.

Because of the incomplete conversion rate of FFPE samples with the EZ Std kit, we checked the reproducibility of the S5 score on a subset of freshly cut FFPE sections, comparing the new pyrosequencing results to the original results. Repeatability of the S5 classifier was good with only three values out of 81 (3.70%) outside the limits of agreement (mean of differences between original and new sections = −3.656, n = 81, Supp. Figure S2). The conversion rate, although inadequate, did not affect the reproducibility of the S5 assay.

### 3.4 Performance of S5 on FFPE biopsies

**FIGURE 6:**
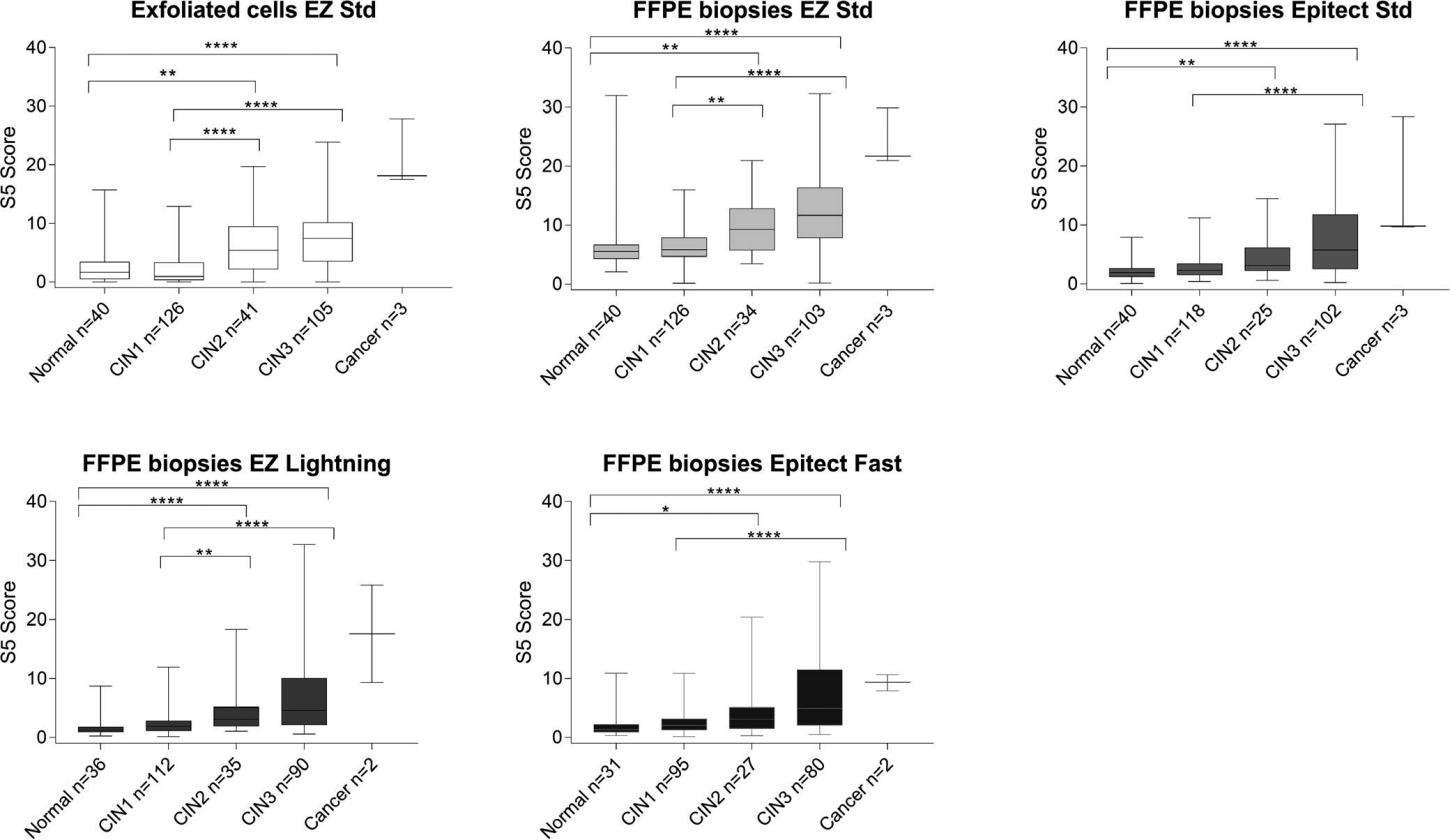
Boxplot of S5 classifier methylation levels by histological groups for all conversion kits. For the exfoliated cells (reference standard) there were significant differences between normal tissue and CIN2 (*p* = 0.009), normal vs CIN3 (*p* < 0.0001), CIN1 vs CIN2 (*p* < 0.0001) and CIN2 vs CIN3 (*p* < 0.0001). The same comparisons were significantly different for the EZ DNA Methylation kit (EZ Std) and EZ DNA Methylation‐Lightning (EZ Lightning). However, for both Epitect bisulfite kits (Standard and Fast) the differences between CIN1 and CIN2 were no longer significant. The cancer group was not tested due to its sample size. *: *p* < 0.05, **: *p* < 0.001, ***: *p* < 0.0005, ****: *p* < 0.0001. Only significant comparisons are represented. The top of boxes represents the upper quartile, bottom the lower quartile and line the median. The whiskers expand to the minimum and maximum values

S5 performed well at discriminating between <CIN2 and CIN2+ samples on the FFPE biopsies for all four kits (Figure 3.). Their methylation values were significantly higher in CIN2+ than in <CIN2 samples (Mann–Whitney tests, *p* < 0.0001). Figure 6 shows the S5 classifier by histological grade. For the exfoliated cells and FFPE material converted with EZ Std and EZ Lightning, the four histological grades (normal, CIN1, CIN2, and CIN3+) had distinct methylation levels as expected (Kruskal–Wallis tests with Dunn's multiple comparisons tests adjusted p values). However, the Epitect Std and Fast kits were not able to provide separation of CIN1 from CIN2 (*p* = 0.0919 for EZ Std and *p* = 0.6840 for EZ Fast) using our pyrosequencing assays.

For all the kits tested, there was a significant trend of increasing methylation levels with a severity of the disease (Cuzick tests for trend, *p* < 0.0001, Figure 6.). The normal and CIN1 samples showed the lowest level of methylation, CIN2 an intermediate level, and CIN3 and cancers the highest.

**FIGURE 7:**
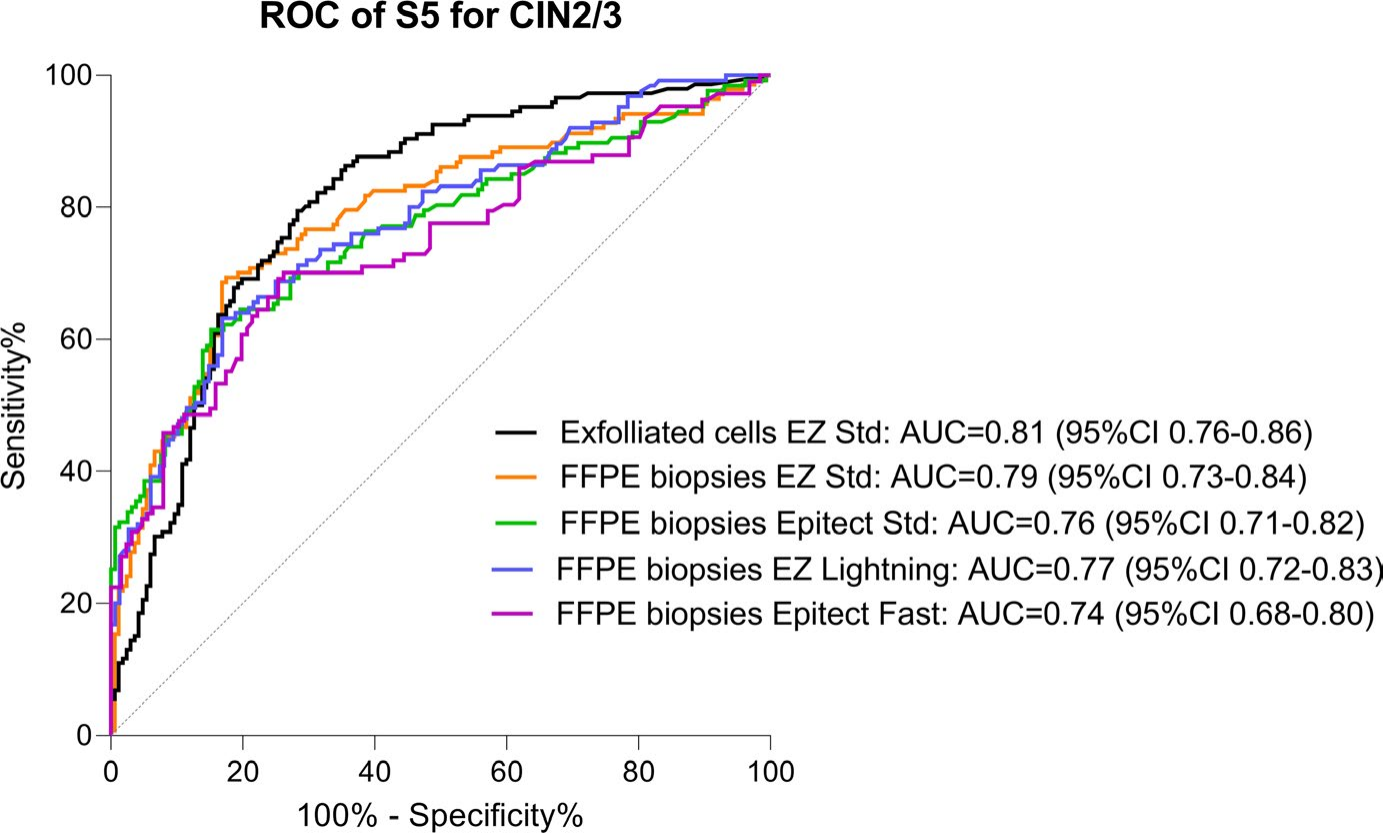
ROC curve of the performance of S5 classifier for CIN2/3 (excluding the 3 cancers) on exfoliated cells and FFPE biopsies bisulfite converted with four different kits. The AUC for S5 on FFPE biopsies was not statistically significantly different from the AUC of S5 calculated on exfoliated cells (DeLong's tests). AUC: area under the ROC curve, CI: confidence interval

All the conversion kits tested on FFPE material performed equally well to detect pre‐cancerous CIN2/3 cases. Figure 7. shows the area under the curve (AUC) of the S5 classifier for the detection of CIN2/3. As expected, the AUC was the highest for the exfoliated cells (AUC =0.81, 95%CI 0.76–0.86, Table 2). The AUCs of S5 from FFPE material were slightly lower but none were significantly different than the AUC of the exfoliated cells (DeLong's tests, Table 2). This confirmed that the S5 methylation classifier is robust in both types of samples and when using various bisulfite conversion kits.

**TABLE 2:** Comparison of AUC, sensitivity, specificity PPV and NPV for detecting CIN2/3 between cells and FFPE biopsies using four different bisulfite conversion kits. For the FFPE biopsies, sensitivities and specificities were calculated at the validated cut‐off of 0.8 for exfoliated cells and at adjusted cut‐offs to provide at least 85% sensitivity

	AUC (95% CI)	*p*‐value	DeLong test *p*‐value	Cut‐off	% Sensitivity (95% CI)	% Specificity (95% CI)	% PPV (95% CI)	% NPV (95% CI)
S5 exfoliated cells Zymo Std	0.81 (0.76–0.86)	<0.0001	Reference	0.80	93.96 (88.84–97.20)	43.37 (37.71–51.27)	59.83 (56.44–63.12)	88.89 (80.58–93.91)
S5 FFPE biopsies Zymo Std	0.79 (0.73–0.84)	<0.0001	0.5849	0.80	99.29 (96.08–99.98)	1.81 (0.37–5.19)	46.03 (45.41–46.65)	75.0 (23.99–96.61)
				5.20 (0.8 + 4.4)	88.57 (82.10–93.32)	41.57 (33.98–49.46)	56.11 (52.60–59.56)	81.18 (72.44–87.62)
				5.72^a^	85.00 (77.99–90.47)	50.60 (42.75–58.44)	59.20 (55.07–63.21)	80.00 (72.40–85.92)
S5 FFPE biopsies Epitect Std	0.76 (0.71–0.82)	<0.0001	0.2578	0.80	96.15 (91.25–98.74)	9.49 (5.41–15.17)	46.64 (45.12–48.16)	75.00 (52.83–88.93)
				1.76^a^	85.38 (78.12–90.97)	38.61 (30.98–46.76)	53.37 (49.80–56.89)	76.25 (66.97–83.56)
S5 FFPE biopsies Zymo Lightning	0.77 (0.72–0.83)	<0.0001	0.3772	0.80	99.21 (95.69–99.98)	11.49 (6.84–17.75)	49.03 (47.53–50.53)	94.44 (69.64–99.21)
				1.47^a^	85.83 (78.53–91.38)	43.92 (35.78–52.31)	56.77 (52.83–60.63)	78.31 (69.40–85.18)
S5 FFPE biopsies Epitect Fast	0.74 (0.68–0.80)	<0.0001	0.1004	0.80	96.33 (90.87–98.99)	9.52 (5.02–16.05)	47.95 (46.26–49.63)	75.00 (49.91–90.03)
				1.42^a^	86.24 (78.32–92.09)	38.10 (29.59–47.17)	54.65 (50.76–58.49)	76.19 (65.55–84.33)

^a^ Adjusted cut‐off to provide at least 85% sensitivity.

S5 on the exfoliated cells specimens had a sensitivity of 93.96%, a specificity of 43.37%, positive predictive value (PPV) of 59.82%, and a negative predictive value (NPV) of 99.89% at the pre‐defined cut‐off of 0.8 ^7^ to detect CIN2/3 cases (Table 2). At the same cut‐off, S5 testing of the FFPE material converted with Zymo Std had greater sensitivity (99.29%), but a much lower specificity (1.81%). This discrepancy was due to the higher S5 values obtained with the FFPE material and the Zymo Std kit because of the incomplete conversion rate. Adjusting the cut‐off to 5.2 to take the increased methylation values into account (by adding the 4.4 difference to the pre‐defined cut‐off of 0.8), the sensitivity became 88.57% and the specificity 41.57% for detecting CIN2+ in the FFPE biopsies as well as an improvement in both PPV and NPV (46.03% to 56.11% and 75.00% to 81.18%, respectively) (Table 2). At the pre‐defined cut‐off of 0.8 the other three bisulfite conversion kits all had high sensitivities, but low specificities despite having similar bisulfite conversion rate to the exfoliated cells. The 0.8 cut‐off gave a PPV of at least 45% and NPV of at least 75% for all kits (Table 2). Adjusting the cut‐off individually for an 85% sensitivity, the specificity was 50.60% for Zymo Std, 38.61% for Epitect Std, 43.92% for Zymo Lightning, and 38.10% for Epitect Fast (Table 2). Adjusting the cut‐off to give 85% PPV gives an NPV of 65.82% for Zymo Std, 65.63% for Epitect Std, 64.65% for Zymo Lightening, and 62.18% for Epitect Fast.

### 3.5 Experiments to improve the conversion rate for the EZ DNA Methylation kit

Various ways to reduce the discrepancy between methylation values in exfoliated cells and FFPE material have been proposed. One of them is to heat the samples at a high temperature or in a buffer of a high pH just before starting the conversion process. We did get a slight improvement with this method when we tested 23 samples using the EZ Std kit. Efficiency was 85% compared to 82% with the original method (Wilcoxon matched pairs signed‐rank test, *p* = 0.0449, Supp. Table S3), but we were still not close to the efficiency obtained with the other conversion kits. We also performed two rounds of conversion. The elution of the first conversion was used as an input for the second round of conversion. With this method, we obtained an average conversion efficiency of 81%, similar to the original method. Finally, we tried three rounds of denaturation at 95°C as used by the Epitect Std kit. We did not obtain any significant improvement with an average efficiency of 81%.

## 4 DISCUSSION

Good epigenetic biomarkers should not only have high sensitivity and specificity, but also be applicable to a variety of sample types, such as fresh or fixed materials, cells, tissue or urine. In this study, we tested the performance of the S5 methylation classifier on 315 paired samples consisting of exfoliated cell samples (reference standard) and FFPE biopsies (166 <CIN2 and 149 CIN2+). In addition, we tested the performance of four different bisulfite conversion kits on the FFPE material. We chose the Zymo EZ DNA Methylation kit (EZ Std) which was the reference kit since it was used to develop the S5 classifier, the Zymo EZ DNA Methylation‐Lightning (EZ Lightning), the Qiagen Epitect Bisulfite Kit (Epitect Std), and the Qiagen Epitect Fast Bisulfite Kit (Epitect Fast).

We showed that the S5 risk scores had comparable AUCs when performed on FFPE biopsies with various bisulfite conversion kits to the AUC obtained from exfoliated cells. The ROC performance of the S5 classifier was not affected by the higher methylation values obtained in the FFPE samples with the EZ Std kit. The AUC of the S5 classifier on the FFPE biopsies was 0.79 with the EZ Std kit, 0.76 with the Epitect Std, 0.77 with the EZ Lightning, and 0.74 with the Epitect Fast kit. None of these values were significantly different from the AUC obtained from the exfoliated cells with the EZ Std kit (0.81). However, using the pre‐defined cut‐off of 0.8, the S5 classifier on the FFPE material bisulfite converted DNA had very low specificity with all the kits, especially with the EZ Std kit. It is clear that new cut‐offs are required to better balance the trade‐off between sensitivity and specificity in FFPE samples. To reflect the higher methylation values obtained in FFPE samples, the S5 cut‐off of 0.8 was provisionally adjusted to 5.2 for the EZ Std kit. At this cut‐off, the sensitivity became 89% for a 42% specificity, values that were similar to those obtained on the exfoliated cells. At the 0.8 cut‐off, the other three bisulfite conversion kits showed a higher sensitivity than the exfoliated cells samples, but also a much lower specificity. The specificity for these kits can be improved by increasing the cut‐off to a higher value while still retaining a sensitivity of 85%.

Despite the elevated methylation levels in the FFPE samples bisulfite converted with EZ Std, the reproducibility of the S5 classifier on these samples was good as indicated by a very small bias (−3.356) and a narrow limit of agreement on the Bland–Altman plot. This is important since it indicates that the assay is technically robust even when using a conversion kit that does not fully convert the unmethylated cytosines into uracils. Newly cut sections were used for this experiment meaning that not only the PCR and pyrosequencing reactions were different from the original assay, but also the DNA extraction and bisulfite conversion steps.

Artificially high methylation levels are expected in FFPE material due to inter‐strand, protein‐DNA, and histone‐DNA crosslinks caused by the formaldehyde fixation.^12^ This causes incomplete DNA denaturation during bisulfite conversion that could vary by genomic location. The bisulfite reaction is then unable to reach the unmethylated cytosines and convert them to uracil, leading to incomplete DNA conversion. Variations in methylation levels are further compounded by each kit having slightly different reaction chemistries, hence despite each manufacturer stating efficiencies of >99% this was almost never the case in our testing. A way to reverse the effect of formaldehyde on the tissue is to heat the DNA before bisulfite conversion.^12^, ^23^ In our study, although we did heat the samples at 90°C for an hour, the bisulfite conversion efficiency of the EZ Std kit (73%) was not as good as the conversion rate obtained from exfoliated cells (95%). Consequently, we observed an average increased value for the S5 classifier of 4.4 in the FFPE samples. Nonetheless, there was a significant correlation for S5 between the paired samples.

Various ways to reduce the discrepancy between methylation values in exfoliated cells and FFPE material have been proposed. We tested three methods and only one of them slightly improved the conversion efficiency rate. Heating the samples at 95°C just before starting the conversion process led to an increased efficiency rate of 3% compared to the original method. Wen et al. ^24^ showed a good methylation agreement between paired fresh frozen and FFPE samples using this method. A major difference between their study and ours is the age of the samples. Our samples were between 10 and 13 years old. The pH of the lysis buffer also seems to be important. Extracting DNA from FFPE in an alkaline buffer (0.1 M NaOH) provided a greater amount of good quality DNA compared to acidic buffers.^23^ This option was not tested since we tested all the kits from the same DNA extraction. These tests highlight the need for further optimization of each kit with each sample material to ensure the most accurate outcome. One of the simplest ways to account for the variation in methylation levels is to alter the cut‐off point to achieve the best sensitivity and specificity or PPV and NPV depending on the aims of the test.

In conclusion, consistent methylation results can be obtained from FFPE material. The S5 classifier has been validated for detecting high‐grade precancers missed by other methods and can also predict which precancers are likely to progress.^3^, ^25^, ^26^ Thus it can be used to prevent cervical cancer by identifying and treating cervical precancerous lesions. The S5 test performed as well on FFPE material as on exfoliated cells but an adjustment for background noise was required. We conclude that FFPE biopsies can be used successfully to accurately diagnose women with CIN2+ samples. The choice of the kit to bisulfite converts the samples made little difference to the sensitivity of the S5 assay, but specificity for FFPE samples was reduced in all the tested kits. Despite the fact that the most elevated background methylation levels were obtained with the Zymo EZ DNA methylation kit on the FFPE material, we can still recommend it as a kit to bisulfite convert samples. However, it is necessary to take into consideration the need for background adjustment.

## 5 ETHICS APPROVAL AND CONSENT TO PARTICIPATE

Ethics no. 05/Q0406/57 given by the Hammersmith and Queen Charlotte's & Chelsea Research Ethics Committee.

## 6 CONSENT FOR PUBLICATION

Not applicable.

## CONFLICT OF INTERESTS

The authors declare that they have no competing interests.

## AUTHORS’ CONTRIBUTIONS

The study plan was conceived by CR, BN, and AL. SB and RB performed the laboratory work. CR and MP analyzed, interpreted the data, and wrote the manuscript. BN, AL, and JC were major contributors in writing the manuscript. All authors read and approved the final manuscript.

## Supporting information

Fig S1

Fig S2

Table S1

Table S2

Table S3

## Data Availability

The data that support the findings of this study are available on request from the corresponding author. The data are not publicly available due to privacy or ethical restrictions.
