# Supplementary material for: Consistency of the S5 DNA methylation classifier in formalin‐fixed biopsies versus corresponding exfoliated cells for the detection of pre‐cancerous cervical lesions
Source: Cancer Med. 2021 Mar 12;10(8):2668–79. doi: 10.1002/cam4.3849 (PMC8026949; doi:10.1002/cam4.3849)
Supplement: Supplementary file 1 — Fig S1 [file CAM4-10-2668-s003.docx]

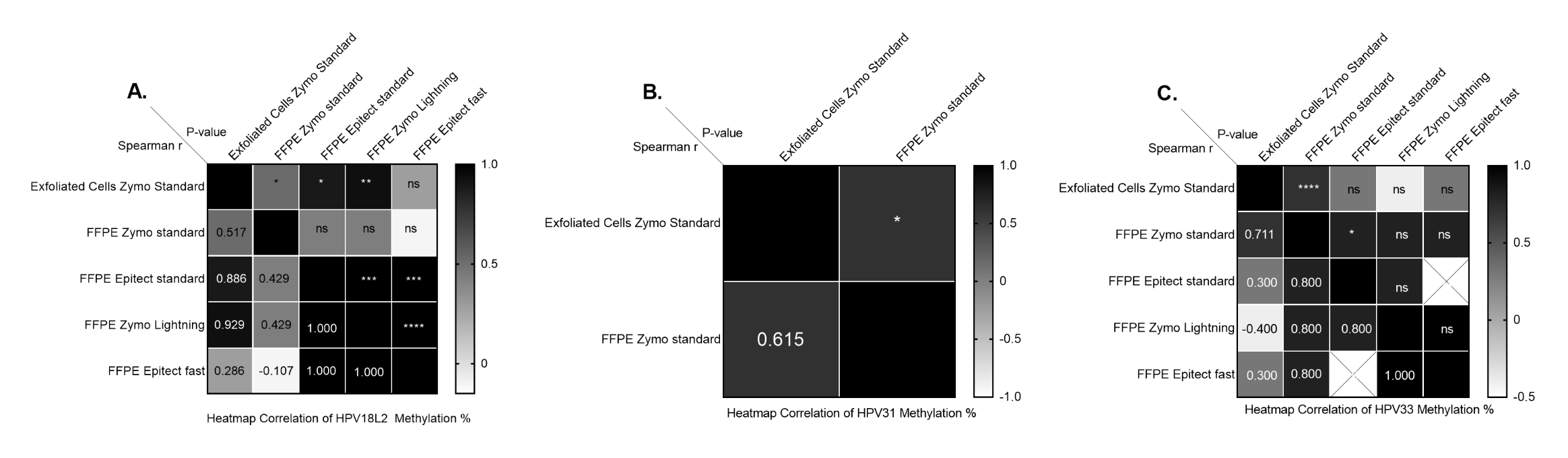


**Supplementary Figure 1.**

Heatmap of the correlation of **A.** HPV18L2, **B.** HPV31 and **C.** HPV33 gene methylation levels obtained from exfoliated cells and FFPE material. DNA from the exfoliated cells was converted with the Zymo EZ DNA Methylation kit (Exfoliated Cells Zymo Standard). Four bisulfite conversion kits were used on the FFPE material: Zymo EZ DNA Methylation (FFPE Zymo Standard), Qiagen Epitect Bisulfite kit (FFPE Epitect Standard), Zymo EZ DNA Methylation-Lightning (FFPE Zymo Lightning) and Qiagen Epitect Bisulfite Fast kit (FFPE Epitect Fast). The Spearman’s correlation coefficients are given below the diagonal and p-values above (ns :p>0.05, *: p<0.05, **: p<0.01, ***: p<0.001, ****: p<0.0001). Crossed boxes represent where there was insufficient data to calculate correlations.
