# Supplementary material for: Consistency of the S5 DNA methylation classifier in formalin‐fixed biopsies versus corresponding exfoliated cells for the detection of pre‐cancerous cervical lesions
Source: Cancer Med. 2021 Mar 12;10(8):2668–79. doi: 10.1002/cam4.3849 (PMC8026949; doi:10.1002/cam4.3849)
Supplement: Supplementary file 2 — Fig S2 [file CAM4-10-2668-s001.docx]

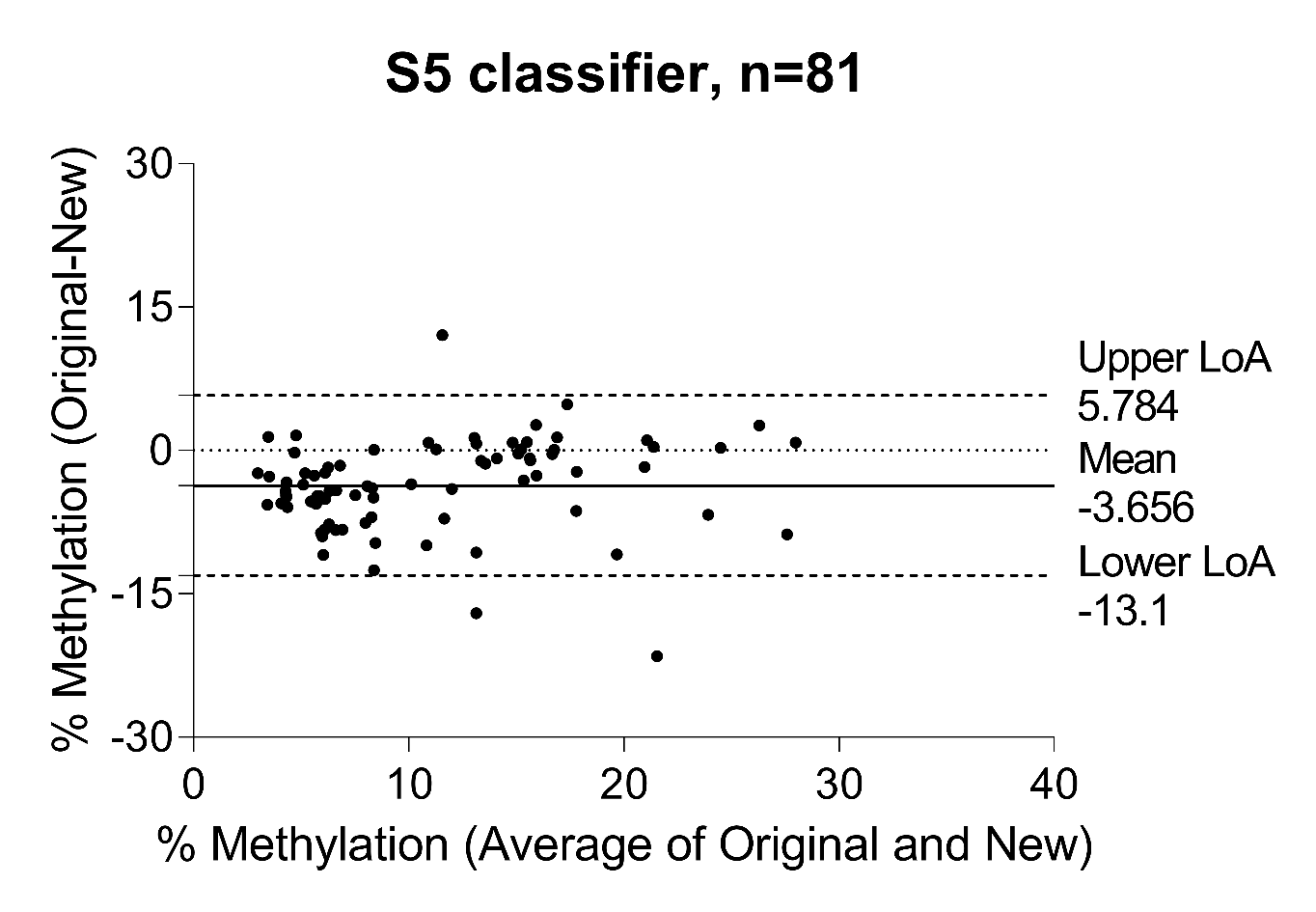


**Supplementary Figure 2.** Assay repeatability for the S5 classifier on FFPE material bisulfite converted with the EZ Std kit. Bland-Altman plot shows the repeatability of the methylation levels of 81 original FFPE sections compared to newly cut sections (new DNA extraction and new DNA bisulfite conversion). The difference of methylation value of the original and the new sections is plotted against their mean. Only 3 samples fell outside the limits of agreement indicating a good repeatability. LoA: Limit of Agreement defined as the mean difference ± 1.96 SD of differences.
