# Supplementary material for: Consistency of the S5 DNA methylation classifier in formalin‐fixed biopsies versus corresponding exfoliated cells for the detection of pre‐cancerous cervical lesions
Source: Cancer Med. 2021 Mar 12;10(8):2668–79. doi: 10.1002/cam4.3849 (PMC8026949; doi:10.1002/cam4.3849)
Supplement: Supplementary file 3 — Table S1 [file CAM4-10-2668-s004.docx]

|  | EZ Std | Epitect Std | EZ Lightning | Epitect Fast |
| --- | --- | --- | --- | --- |
| DNA protect buffer |  | tetrahydrofurfuryl alcohol |  | tetrahydrofurfuryl alcohol |
| Conversion reagent | sodium metabisulphite + sodium hydroxide | sodium metabisulphite | Amonium bisulfite | ammonium hydrogensulphite |
| Denaturation | 15 min 37ºC in sodium hydroxide | 5 min 95ºC | 8 min 98ºC | 5 min 95ºC |
| Incubation | 16 hours 50ºC | 25 min 60ºC | 60 min 54ºC | 10 min 60ºC |
| Denaturation |  | 5 min 95ºC |  | 5 min 95ºC |
| Incubation |  | 85 min 60ºC |  | 10 min 60ºC |
| Denaturation |  | 5 min 95ºC |  |  |
| Incubation |  | 175 min 60ºC |  |  |
| Binding to column | Guanidinium chloride | Guanidine thiocyanate + carrier RNA | Guanidinium chloride | Guanidine thiocyanate + carrier RNA |
| Desulphonation | Sodium hydroxide, ehtanol, propanol | Sodium hydroxide | Sodium hydroxide, ehtanol, propanol | Sodium hydroxide |

**Supp. Table 1.** Composition and procedures of the four bisulfite conversion kits used in the study.
