# Supplementary material for: Consistency of the S5 DNA methylation classifier in formalin‐fixed biopsies versus corresponding exfoliated cells for the detection of pre‐cancerous cervical lesions
Source: Cancer Med. 2021 Mar 12;10(8):2668–79. doi: 10.1002/cam4.3849 (PMC8026949; doi:10.1002/cam4.3849)
Supplement: Supplementary file 4 — Table S2 [file CAM4-10-2668-s005.docx]

* Bonferoni correction, α=0.0125

**Supp. Table 2.** Bisulfite conversion efficiency as assessed by quantitative methylation specific PCR of the β-actin gene, comparing FFPE biopsy DNA to the reference exfoliated cell DNA, using four different bisulfite conversion kits.
