# Supplementary material for: Consistency of the S5 DNA methylation classifier in formalin‐fixed biopsies versus corresponding exfoliated cells for the detection of pre‐cancerous cervical lesions
Source: Cancer Med. 2021 Mar 12;10(8):2668–79. doi: 10.1002/cam4.3849 (PMC8026949; doi:10.1002/cam4.3849)
Supplement: Supplementary file 5 — Table S3 [file CAM4-10-2668-s002.docx]

|  | Original EZ Std | Higher temperature | Double conversion | Multiple denaturations |
| --- | --- | --- | --- | --- |
| N | 23 | 23 | 23 | 23 |
| Minimum | 54.95 | 38.27 | 26.00 | 33.10 |
| Maximum | 92.60 | 94.27 | 96.14 | 98.25 |
| Average | 82.30 | 84.57 | 80.62 | 80.64 |
| Std Deviation | 8.71 | 12.41 | 19.47 | 16.82 |
| Wilcoxon matched-pairs signed rank test p-values | Ref | 0.045 | 0.964 | 0.988 |

**Supp. Table 3.** Attempts at improving conversion rate of FFPE material with the EZ DNA Methylation (EZ Std) kit.
